# Development of New Antibodies and an ELISA System to Detect the Potato Alkaloids α-Solanine and α-Chaconine

**DOI:** 10.3390/foods12081621

**Published:** 2023-04-12

**Authors:** Kohki Okada, Kano Matsuo

**Affiliations:** 1 Department of Medical Technology and Sciences, Faculty of Health Sciences, Kyoto Tachibana University, Kyoto 607-8175, Japan; 2 Graduate School of Health Sciences, Kyoto Tachibana University, Kyoto 607-8175, Japan

**Keywords:** α-chaconine, α-solanine, enzyme-linked immunosorbent assay, food poisoning, food testing, glycoalkaloid, laboratory medicine, potato alkaloids, solanidine

## Abstract

Food poisoning can be caused by the potato alkaloids α-solanine (SO) and α-chaconine (CHA). Therefore, this study aimed to establish new enzyme-linked immunosorbent assays (ELISAs) for detecting these two toxins in biological samples and potato extracts. Two antibodies that bind to solanidine, a chemical compound found in both SO and CHA, were newly developed, and two types of ELISAs (Sold1 ELISA and Sold2 ELISA) were constructed. We measured SO and CHA diluted in phosphate-buffered saline (PBS), serum, and urine. The detection performance of the two ELISAs for SO and CHA in PBS was higher than in serum and urine, and the sensitivity of Sold2 ELISA was lower than that of Sold1 ELISA. Thus, we used these ELISAs to measure SO and CHA in potato part extracts and found that potato sprouts contained approximately 80-fold more SO and CHA than tubers and 8-fold more SO and CHA than peels. Although the detection sensitivity of SO and CHA depends on the sample types, these ELISAs may be effective as future clinical and food testing methods after further improvements.

## 1. Introduction

α-solanine (SO) and α-chaconine (CHA) are natural toxins that are mainly produced in potatoes in response to pest attacks [1] and comprise approximately 95% of the total glycoalkaloid (GA) content in potatoes. The oral ingestion of these toxins often causes food poisoning in humans [2]; according to a previous report, ingestion of more than 1 mg of GAs per kg of body weight is toxic to humans [3]. Symptoms of GA food poisoning include vomiting, diarrhea, cardiac dysrhythmia, and inflammation and pain in the joints [4]. Although the concentration of GAs varies among potato species, it generally ranges from 0.4 to 1000 μg/g in commercial potatoes [5]. GAs are more abundant in the peel and sprout of potatoes than in the tuber, and light exposure and long-term storage further increase the GA content in these parts [6,7,8]. Furthermore, only a few percent of these toxins are eliminated during cooking, such as boiling or frying [7]. Given the global distribution of potatoes, the occurrence of GA food poisoning continues to be inevitable, however, efforts should be taken to reduce the number of fatal patients.

The development of food testing methods is important to avoid the consumption of potatoes rich in GAs. In 1994, Stanker et al. succeeded in developing the first monoclonal antibody against GAs [9]. The limit of detection (LOD) for GAs measured using an enzyme-linked immunosorbent assay (ELISA) kit constructed with this antibody was approximately 70 ng/mL [10]. Simultaneously, the measurement of GAs in potatoes using high-performance liquid chromatography (HPLC) was developed, and the LODs of SO and CHA calculated using this method were 1.2 ng/mL and 1.3 ng/mL, respectively [8,11]. Recently, the detection performance of GAs in potatoes has been significantly improved using the combination of HPLC and mass spectrometry [12]. Although several methods for detecting GAs in potato extracts have been reported, few studies have detected GAs in biological samples, such as serum and urine, which is critical for diagnosing patients with GA food poisoning. Hellenäs et al. were the first to successfully detect potato GAs in serum at a LOD of 0.3 ng/mL using HPLC [13]. Mensinga et al. also used HPLC to measure the changes in human serum GA concentrations over time, which were detectable in the range of 0.5–50 ng/mL [14]. Despite several components in serum, HPLC can efficiently detect GAs in serum. However, in recent years, few studies have developed an efficient detection method for potato-derived SO and CHA in biological samples.

The effects of eating stale potatoes should not be underestimated, as there have been some cases of deaths due to GA food poisoning [15]. However, in previous case reports, the diagnosis of potato food poisoning was based on the fact that the patients consumed old potatoes and suffered from clinical symptoms and not on the measurement of GAs in their biological samples [16]. Few medical institutions diagnose potato food poisoning by measuring GAs. As many hospitals are not equipped with HPLC or mass spectrometry instruments, it is difficult to effectively and rapidly measure the GA contents in the biological samples of patients with GA food poisoning. If there is an established laboratory method for measuring GAs in biological samples, physicians or medical practitioners can determine whether the treatment of the patients is appropriate and whether any GAs are retained in the body. In order to develop a laboratory method for detecting GAs, it is important to generate antibodies that can reliably capture GAs. By using superior antibodies as reagents, immunological assays can be constructed or incorporated into automated analyzers in medical facilities.

In the present study, we aimed to identify new antibodies that can bind to both SO and CHA and construct two types of ELISAs using them. Further, we evaluated the detection performance of these ELISAs for SO and CHA in two biological samples (serum and urine) and potato part extracts to verify their usefulness as clinical and food testing methods. We found that although the ELISAs established in our study had low detection sensitivity for the serum and urine samples, they can be used as clinical diagnosis methods after certain improvements. Further, as they can efficiently measure the SO and CHO concentrations in potato extracts, they also have the potential to be used as food testing methods.

## 2. Materials and Methods

### 2.1. Reagents

SO and CHA powders were purchased from Sigma-Aldrich Co., LLC (Tokyo, Japan). Horseradish peroxidase (HRP)-labeled goat anti-rabbit polyclonal IgG (H + L) antibodies were obtained from Funakoshi Co., Ltd. (Tokyo, Japan). Commercial serum and urine samples of healthy volunteers were obtained from Cosmo Bio Co., Ltd. (Tokyo, Japan). Bio-Safe Coomassie stain, Clarity Western ECL substrate kits, Precision Plus Protein Dual Color Standards, Trans-Blot Transfer Packs, and 0.2 μm pore-size nitrocellulose membranes were obtained from Bio-Rad Laboratories, Inc. (Hercules, CA, USA). A 96-well ELISA plate H was purchased from Sumitomo Bakelite Co., Ltd. (Tokyo, Japan). An HRP labeling kit was obtained from Dojindo Laboratories (Kumamoto, Japan). Blocking One and dimethyl sulfoxide (DMSO) were purchased from Nacalai Tesque, Inc (Kyoto, Japan). The Prominence LC-2010 HPLC system and the reversed-phase chromatography (RPC) column Shim-pack GIST C18-AQ were purchased from Shimadzu Corporation (Kyoto, Japan). Freund’s complete adjuvant (FCA), Freund’s incomplete adjuvant (FIA), o-phenylenediamine dihydrochloride (OPD) tablets, 2-mercaptoethanol (2-ME), and all other reagents were obtained from Wako Pure Chemical Industries, Ltd. (Tokyo, Japan) and Wakenyaku Co., Ltd. (Kyoto, Japan).

### 2.2. Sample Preparation

**Figure 1:**
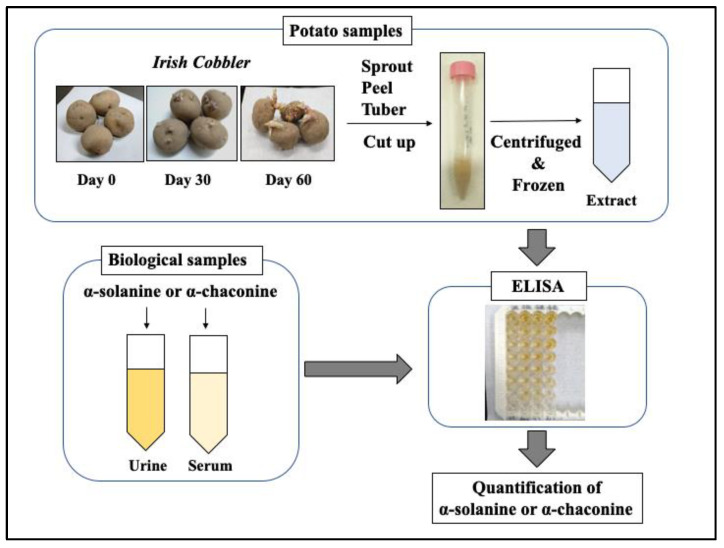
Preparation procedure for each sample. The upper figure shows the preparation process for the potato extract samples. The lower left illustration indicates the preparation of biological samples. Enzyme-linked immunosorbent assays (ELISAs) were conducted using these samples as shown in the lower right image.

SO and CHA powders were individually dissolved in 10% DMSO and used in subsequent experiments. The prepared SO and CHA solutions were diluted in 10 mM phosphate-buffered saline (PBS, pH 7.4), serum, or urine and were used as assay samples. In addition, extracts from potatoes were also used as samples. Potatoes (Irish Cobbler, total *n* = 12) were purchased from a supermarket and kept under fluorescent light (380–400 nm wavelength) at 22 °C during the experimental period. Their components were extracted immediately after purchase (Day 0, *n* = 4), after 30 days (Day 30, *n* = 4), and after 60 days (Day 60, *n* = 4). The extracted parts were sprouts (Day 30 and 60), peels (Day 0, 30, and 60), and tubers (Day 0, 30, and 60). Each part was removed from the potatoes (in particular, tubers were carefully removed within 0.5 cm from the peels), weighed, and mixed with 5 mL of 10% DMSO in 15 mL polycarbonate tubes. The parts were homogenized using a 150 Homogenizer (Fisher Scientific Co LLC, Waltham, MA, USA) in the tubes and centrifuged at 1191× *g* for 30 min at 4 °C. These supernatants were used as the assay samples and stored at −80 °C until use. The sample preparation protocol is summarized in Figure 1.

### 2.3. Development of Solanidine-Binding Antibodies

**Figure 2:**
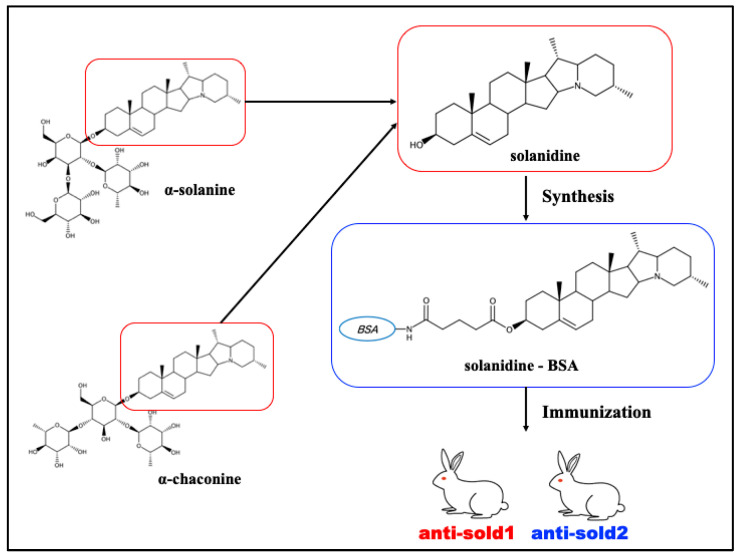
The development procedure for developing solanidine antibodies. The two illustrations on the left show the molecular structures of α-solanine and α-chaconine. These two toxins have the chemical structure of solanidine (the red box on the right). By immunizing two rabbits with a complex of solanidine conjugated with bovine serum albumin (BSA) (solanidine-BSA, the blue box on the right) as the immunogen, two kinds of polyclonal antibodies (anti-sold1 and anti-sold2) were obtained.

The development of antibodies that bind to SO and CHA was completely outsourced to Carbuncle BioScienTec LLC (Kyoto, Japan). This experiment complied with the ARRIVE guidelines (Animal Research: Reporting of In Vivo Experiments) and was approved by the Animal Experiment Committee in Carbuncle BioScienTec LLC. Briefly, solanidine, a chemical compound found in both SO and CHA, was conjugated with bovine serum albumin as an immunogen. Two female rabbits (Japanese White; Nippon Institute for Biological Science; weight, 3.0 kg) were immunized intradermally with 0.5 mg of this immunogen (per rabbit) five times (first time: with FCA, second–fifth time: with FIA) every two weeks. During this period, blood was collected from the ear vein one week after immunization. To obtain the serum, all of these blood samples were left at room temperature (22 °C) for 30 min and then centrifuged at 1191× *g* for 10 min, after which the supernatant was collected. In accordance with a previous report [17], antibody titers in these sera were confirmed by direct ELISA. For the present study, we ensured the collection of sufficient antibody titers, as the results of ELISA performed with 40,000-fold diluted serum after 5 times immunization had previously shown absorbance values of 1.5–2.0 at 450 nm/630 nm wavelengths. Subsequently, blood was drawn from the carotid artery of the rabbits under anesthesia, and 72 mL antiserum was obtained from one rabbit and 69 mL antiserum from the other. Then, rabbit anti-solanidine polyclonal IgGs (anti-sold1 and anti-sold2) were purified from each of the two antisera using an agarose affinity column on which the solanidine-ovalbumin complex was adhered to. The protein concentration of the purified antibodies was quantified using the Lowry method [18]. The procedure for developing these antibodies is summarized in Figure 2. The chemical structures of these compounds were obtained from the PubChem homepage and illustrated using the Ketcher 2.7 software (EPAM Systems Inc. Newtown, PA, USA) [19,20].

### 2.4. SDS-PAGE and Western Blotting

The purified antibodies were detected by sodium dodecyl sulfate-polyacrylamide gel electrophoresis (SDS-PAGE) as described in a previous study [21]. The concentration of all polyacrylamide gels was 12.5%. After SDS-PAGE, proteins were transferred to nitrocellulose membranes using the Trans-Blot Turbo system (Bio-Rad Laboratories, Inc., Hercules, CA, USA). After blocking the membranes with Blocking One, they were incubated at 4 °C for 1 h with 2 μg/mL HRP-labeled goat anti-rabbit polyclonal IgG (H + L). The membranes were then washed for 5 min thrice with 10 mM Tris-HCl buffer (pH 7.4) and 0.9% NaCl (buffer A), twice with buffer A/0.1% Tween 20, and once with buffer A. Subsequently, antibody-bound proteins were detected using a Chemi-Doc^TM^ XRS plus imaging system and Clarity Western ECL substrate (Bio-Rad Laboratories, Inc.).

### 2.5. Construction of Non-Competitive Direct ELISAs

**Figure 3:**
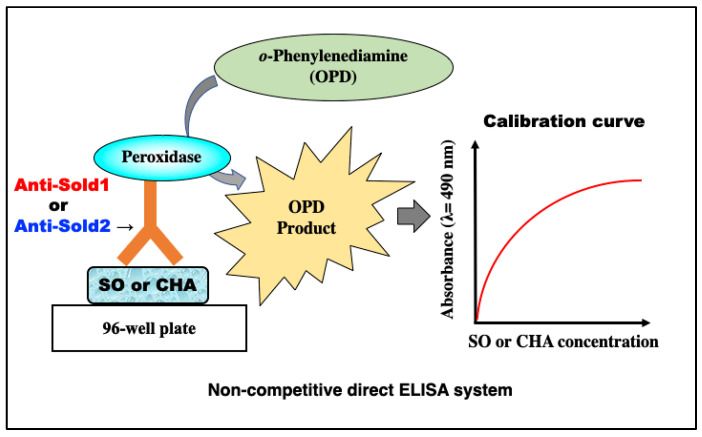
The design of a non-competitive direct ELISA. The left illustration shows the ELISA design used in this study. In this ELISA, the relationship between α-solanine (SO) or α-chaconine (CHA) concentration and the absorbance is denoted by a sigmoid curve, as depicted by the graph on the right.

Prior to ELISA construction, anti-sold1, and anti-sold2 were labeled to HRP (anti-sold1-HRP and anti-sold2-HRP, respectively) using an HRP labeling kit–SH, according to the manufacturer’s protocol. In accordance with a previous report [22], two kinds of non-competitive direct ELISAs were constructed using anti-Sold1-HRP (Sold1 ELISA) and anti-Sold2-HRP (Sold2 ELISA). Briefly, 100 μL of 75 mM carbonate buffer (pH 9.6) was added to each well of a 96-well ELISA Plate H, followed by the addition of 25 μL of sample solutions to the plate and then incubating the plate overnight at 4 °C. After the plate had been washed three times with 10 mM PBS (pH 7.4) and 0.05% Tween 20 (PBS-T), 200 µL of Blocking One was added to each well, and then the plate was incubated for 1 h at 22 °C. The plate was washed with PBS-T again three times, and 100 μL of 2 μg/mL anti-Sold1-HRP or anti-Sold2-HRP diluted with Blocking One was added to each well, followed by incubating the plate for 2 h at 22 °C. Prior to the next plate washing, a chromogenic solution was prepared by dissolving a 5 mg OPD tablet in 10 mL of 0.1 M citrate buffer (pH 5.0). After the plate had been washed three times with PBS-T, 5 μL of 30% H_2_O_2_ was immediately added to the 10 mL chromogenic solutions, and then 100 μL of the mixture solution was added to each well. After 30 min of color development, the reaction was stopped by adding 100 μL of 3 N H_2_SO_4_ to each well. The absorbance of the color reaction was measured at a wavelength of 490 nm using a microplate reader (Bio-Rad Laboratories, Inc.). A schematic diagram of this experiment is illustrated in Figure 3.

### 2.6. HPLC Analysis

In this study, HPLC analysis was performed using the Prominence LC-2010 HPLC system connected to a Shim-pack GIST C18-AQ column based on RPC (Shimadzu Corporation, Kyoto, Japan). Analyses were performed at a room temperature of 22 °C. The flow rate was fixed at 1.0 mL/min, and 10 mM PBS (pH 7.4) was used as the mobile phase. The volume of all samples used for analysis was 100 μL. After sample injection into the HPLC system, a waveform at a wavelength of 210 nm was recorded with a retention time of 30 min. After measurement, the detected waveforms were analyzed using the LabSolutions software version 5.92 provided with the instrument. The lower areas of the waveforms of SO and CHA were calculated using this software to create a calibration curve and to measure the content of SO and CHA in the samples.

### 2.7. Statistical Analysis

All experiments except for the ones that verified the reproducibility of the ELISAs were measured in quintuplicate, and the data are shown as the mean ± standard deviation (SD). The coefficient of variation (CV) was calculated according to the following equation: (SD/mean) × 100%. In accordance with a previous report [23], the LOD was defined as the mean concentration of blank samples (*n* = 16) + 3SD. The recovery of SO and CHA in each ELISA was calculated according to the following equation: recovery = [(final concentration − initial concentration)/added concentration] × 100%. To create the calibration curve, this formula and Spearman’s rank correlation coefficient (R-value) were calculated using the KaleidaGraph software version 5.0.4 (Hulinks Inc., Tokyo, Japan).

## 3. Results

### 3.1. Detection of Purified Polyclonal Antibodies

**Figure 4:**
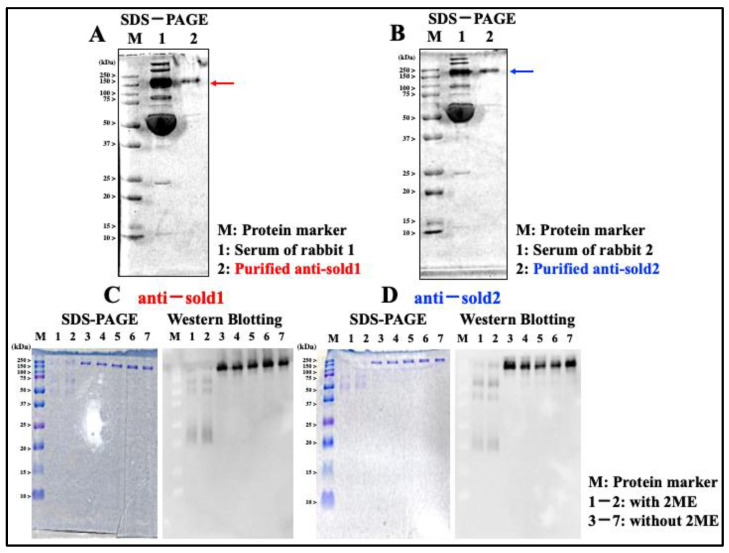
The detection of purified antibodies. (**A**,**B**) The results of sodium dodecyl sulfate-polyacrylamide gel electrophoresis (SDS-PAGE) showing the serum components and antibodies. Lanes 1 and 2 show the bands of serum components and purified antibodies, respectively, (red and blue arrows) obtained from two different rabbits. (**C**,**D**) The results of SDS-PAGE (left panel) and Western blotting (right panel) showing the bands of antibodies with/without 2-mercaptoethanol (2-ME). Lanes 1 and 2 show the bands of purified antibodies with 2-ME obtained from the two different rabbits. Lanes 3–7 show the bands of purified antibodies without 2-ME obtained from the two different rabbits. Lane M in all the pictures indicates a protein marker, and the numbers on the left side of the lane represent the molecular weight (kDa) of each component. The SDS-PAGE results depicted in panels C and D were photographed with a digital camera, and those depicted in the other panels were captured using a ChemiDoc^TM^ XRS plus imaging system (Bio-Rad Laboratories Inc.).

To confirm that the rabbit polyclonal antibodies were successfully purified, SDS-PAGE and Western blotting were performed. Although the serum contained many components (Figure 4A,B, and lane 1), only IgGs were purified by eluting the proteins bound to the affinity column (Figure 4A,B and lane 2). To confirm that the purified protein is IgG, the fragmentation was verified by adding 2-ME to the sample. In the sample without 2-ME, the bands of IgG had a molecular weight of 150 kDa (Figure 4C,D and lanes 3–7); however, in the sample with 2-ME, the bands disappeared, and fragmentation was confirmed by SDS-PAGE and Western blotting (Figure 4C,D and lanes 1–2). We succeeded in purifying anti-sold1 and anti-sold2 and thus proceeded to construct the ELISAs.

### 3.2. Construction of Two ELISAs to Detect SO and CHA

**Figure 5:**
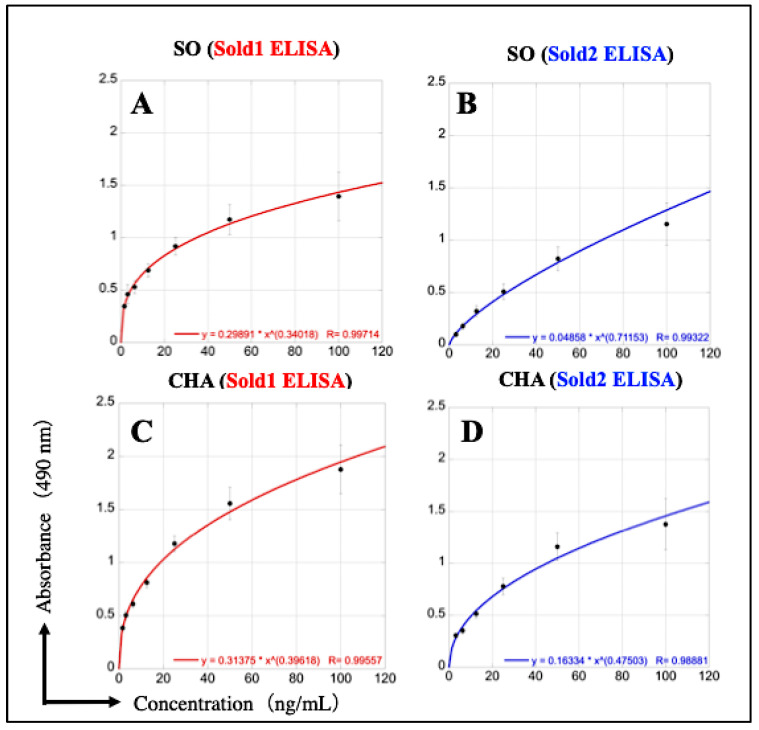
The calibration curves of each ELISA using serial dilutions of the SO and CHA in 10 mm phosphate-buffered saline (PBS, pH 7.4). The blank-subtracted absorbance mean (black circles) ± SD (error bars) values were plotted against SO (**A**,**B**) and CHA (**C**,**D**) concentrations to construct the calibration curves of Sold1 ELISA (**A**,**C**) and Sold2 ELISA (**B**,**D**). In each graph, the *x*-axis indicates the SO or CHA concentrations (ng/mL) and the *y*-axis indicates their absorbance at 490 nm. The samples for all ELISAs were seven two-fold serial dilutions of the SO and CHA standard (1.56–100 ng/mL), using PBS as the diluent and blank. The calibration curves were graphed, and the formula and R-value were calculated using the KaleidaGraph software.

Two non-competitive direct ELISAs were constructed using anti-sold1 and anti-sold2. When the seven two-fold serial dilutions with PBS were measured, Sold1 ELISA showed good calibration curves for SO and CHA in the concentration range of 1.56–100 ng/mL (Figure 5A,C), whereas Sold2 ELISA was able to make calibration curves in the concentration range of 3.12–100 ng/mL (Figure 5B,D). The calculated LODs of SO had better values in the Sold1 ELISA (1.38 ng/mL) than in the Sold2 ELISA (2.95 ng/mL) (Table 1 and Table 2). Similarly, the LODs of CHA were better in the Sold1 ELISA (1.08 ng/mL) than in the Sold2 ELISA (2.76 ng/mL) (Table 1 and Table 2). Furthermore, the detection sensitivity of CHA was slightly higher than that of SO in both of the ELISAs.

### 3.3. Verification of the Repeatability of Sold1 ELISA

**Figure 6:**
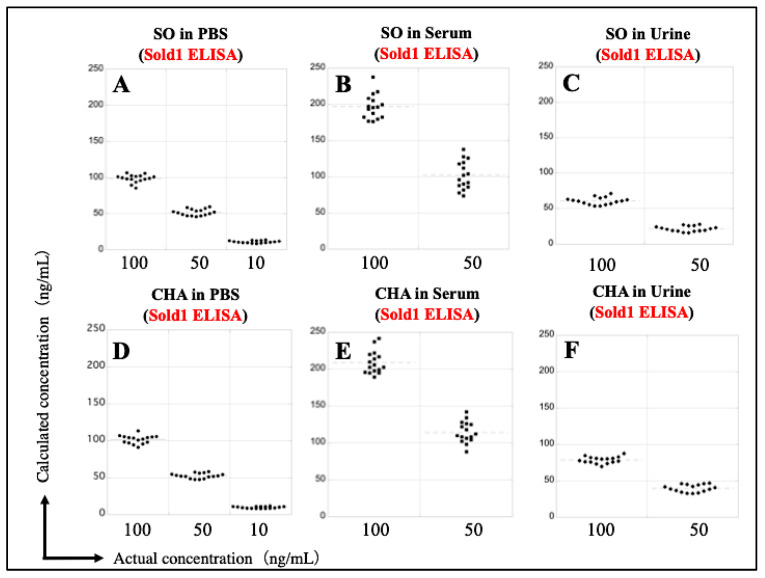
The repeatability of Sold1 ELISA for measuring SO and CHA concentrations in different biological matrices. The Sold1 ELISA was used to simultaneously measure 16 samples of SO (**A**–**C**) and CHA (**D**–**F**), each at concentrations of 10 ng/mL (PBS only), 50 ng/mL, and 100 ng/mL in different biological matrices (PBS: circles, serum: squares, and urine: diamonds). Columns indicate the detection of SO and CHA in PBS (**A**,**D**), serum (**B**,**E**), and urine (**C**,**F**). In the graphs, the *x*-axis indicates the actual concentrations (ng/mL) of the samples prepared by dissolving SO and CHA powders in these matrices and the *y*-axis indicates the SO and CHA concentrations (ng/mL) calculated from the Sold1 ELISA calibration curves (Figure 5A,C). The dotted lines indicate the average of the measurements.

To evaluate the repeatability of Sold1 ELISA, 16 samples each of SO and CHA diluted to 10 ng/mL, 50 ng/mL, and 100 ng/mL with PBS, serum, and urine as solvents were measured. When the SO and CHA samples diluted in PBS were measured, Sold1 ELISA showed excellent CV and simultaneous repeatability (Figure 6A,D and Table 1). However, a non-specific chromogenic reaction was observed in the samples in which SO and CHA were diluted with serum, and the 10 ng/mL samples could not be measured, whereas the calculated concentrations of the 50 ng/mL and 100 ng/mL samples were approximately 2-fold higher than the actual concentrations (Figure 6B,E and Table 1). Then, the same serum samples were deproteinized by adding 10% trichloroacetic acid, after which they were measured by ELISA. Although the non-specific reaction was suppressed by the deproteinization treatment of the serum, the coloration itself was hardly observed. Furthermore, the chromogenic reaction was suppressed in the samples in which SO and CHA were diluted in urine. The 10 ng/mL samples could not be measured, whereas the 50 ng/mL and 100 ng/mL samples showed somewhat lower values than their actual concentrations (Figure 6C,F and Table 1).

### 3.4. Verification of the Repeatability of Sold2 ELISA

**Figure 7:**
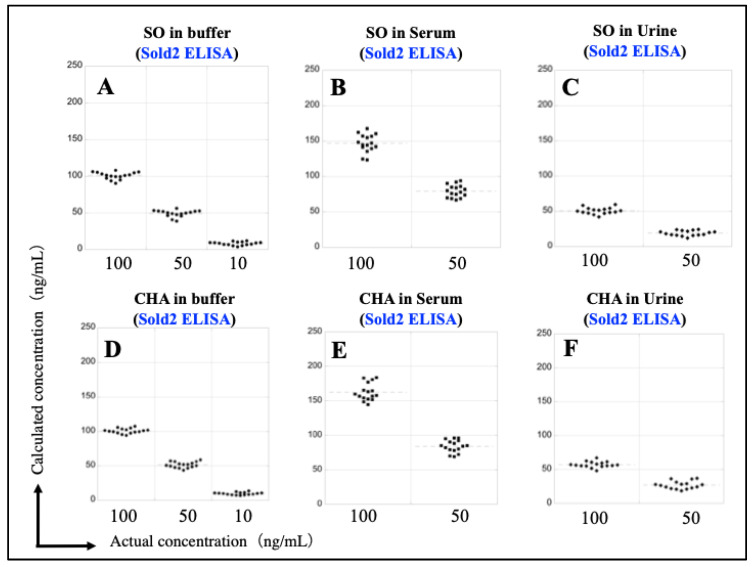
The repeatability of Sold2 ELISA for measuring the SO and CHA concentrations in different biological matrices. Sold2 ELISA was used for measuring 16 equivalent SO (**A**–**C**) and CHA (**D**–**F**) samples with concentrations of 10 ng/mL (PBS only), 50 ng/mL, and 100 ng/mL in different biological matrices (PBS: circles, serum: squares, and urine: diamonds). Columns indicate the detection of SO and CHA in PBS (**A**,**D**), serum (**B**,**E**), and urine (**C**,**F**). In the graphs, the *x*-axis indicates the actual concentrations (ng/mL) of the samples prepared by dissolving SO and CHA powders in these matrices and the *y*-axis indicates the SO and CHA concentration (ng/mL) calculated by Sold2 ELISA calibration curves (Figure 5B,D). The dotted lines indicate the average of the measurements.

The repeatability of Sold2 ELISA was investigated using the same procedure as for Sold1 ELISA. The concentrations of SO and CHA diluted in PBS were accurately measured with only small variations (Figure 7A,D and Table 2). Slightly nonspecific chromogenic reactions were observed in the samples of SO and CHA diluted with serum, with 10 ng/mL not being measurable and 50 ng/mL and 100 ng/mL calculated to concentrations about 1.5 times higher than the actual concentrations (Figure 7B,E and Table 2). The chromogenic reaction was inhibited in the samples of SO and CHA diluted in urine, with 10 ng/mL not being measurable and 50 ng/mL and 100 ng/mL calculated to be about half the actual concentration (Figure 7C,F and Table 2).

### 3.5. Reconstruction of Sold1 ELISA Calibration Curves Using the Dilution Series of SO and CHA in Serum and Urine

**Figure 8:**
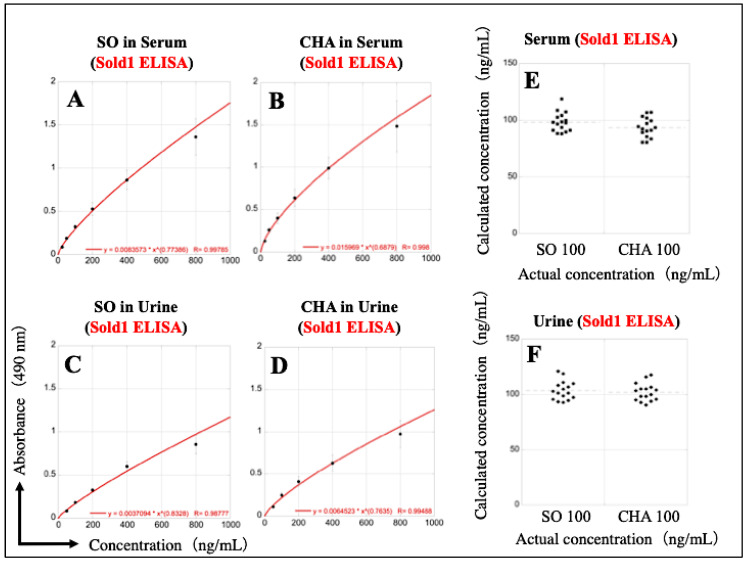
Reconstruction of the calibration curves to improve the accuracy of SO and CHA quantification in serum and urine by Sold1 ELISA. To adjust for the interfering substances present in serum and urine, new calibration curves were constructed for serum (**A**,**B**) and urine (**C**,**D**), based on seven serial two-fold dilutions of SO and CHA (12.5–800 ng/mL), using each biological matrix type as the diluent and blank. The blank-subtracted absorbance mean (black circles) ± SD (error bars) values were plotted against SO (**A**,**C**) and CHA (**B**,**D**) concentrations to construct the calibration curves by Sold1 ELISA. In each graph, the *x*-axis indicates the SO and CHA concentrations (ng/mL) and the *y*-axis indicates their absorbance at 490 nm. The calibration curves were graphed, and the formula and R-value were calculated using the KaleidaGraph software. The repeatability of SO and CHA quantification in serum (**E**) and urine (**F**) was re-evaluated using the new calibration curves, via 16 simultaneous measurements of SO and CHA in these biological matrices (serum: squares, urine: diamonds). In the graphs, the *x*-axis indicates the actual concentrations (100 ng/mL) prepared by the SO and CHA powders diluted in these matrices, and the *y*-axis indicates the SO and CHA concentrations (ng/mL) calculated by Sold1 ELISA reconstructed calibration curves (**A**–**D**). The dotted lines indicate the average of the measurements.

To correctly measure the SO and CHA concentrations in the serum and urine, Sold1 ELISA calibration curves were newly constructed from the measurement results of the seven two-fold dilution series (12.5–800 ng/mL) using serum and urine as their solvents. Good calibration curves of SO and CHA diluted in serum could be made in the concentration range of 25–800 ng/mL (Figure 8A,B). The LOD of SO in serum was 15.25 ng/mL, whereas that of CHA in serum was 13.48 ng/mL (Table 3). Meanwhile, calibration curves of SO and CHA diluted in urine could be constructed in the concentration range of 50–800 ng/mL (Figure 8C,D). The LOD of SO in urine was 30.28 ng/mL, whereas that of CHA in urine was 27.92 ng/mL (Table 3). Based on these calibration curves, 16 samples of 100 ng/mL SO and CHA diluted in serum were re-measured, and the simultaneous repeatability was improved (Figure 8E and Table 3). The repeatability of simultaneous measurements was also improved when 16 samples of 100 ng/mL SO and CHA diluted in urine were re-measured (Figure 8F and Table 3).

### 3.6. Reconstruction of Sold2 ELISA Calibration Curves Using the Dilution Series of SO and CHA in Serum and Urine

**Figure 9:**
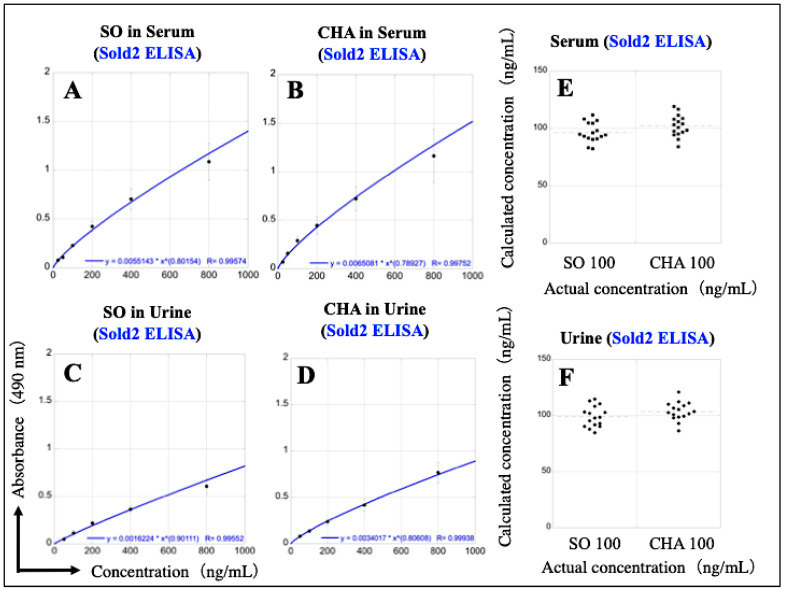
Reconstruction of calibration curves to improve the accuracy of SO and CHA quantification in serum and urine by Sold2 ELISA. To adjust for the interfering substances present in serum and urine, new calibration curves were constructed for serum (**A**,**B**) and urine (**C**,**D**), based on seven serial two-fold dilutions of SO and CHA (12.5–800 ng/mL), using each biological matrix type as the diluent and blank. The blank-subtracted absorbance mean (black circles) ± SD (error bars) values were plotted against SO (**A**,**C**) and CHA (**B**,**D**) concentrations to construct calibration curves by Sold2 ELISA. In each graph, the *x*-axis indicates the SO and CHA concentration (ng/mL) and the *y*-axis indicates their absorbance at 490 nm. The calibration curves were graphed, and the formula and R-value were calculated using the KaleidaGraph software. The repeatability of SO and CHA quantification in serum (**E**) and urine (**F**) was re-evaluated using the new calibration curves, via 16 simultaneous measurements of SO and CHA in these biological matrices (serum: squares, urine: diamonds). In the graph, the *x*-axis indicates the actual concentrations (100 ng/mL) prepared by diluting SO and CHA powders in these matrices, and the *y*-axis indicates the SO and CHA concentrations (ng/mL) calculated by Sold2 ELISA reconstructed calibration curves (**A**–**D**). The dotted lines indicate the average of the measurements.

Similarly, the Sold2 ELISA calibration curves for serum and urine were re-created and verified for simultaneous repeatability. Sold2 ELISA was able to construct calibration curves for SO and CHA diluted in serum in the concentration range of 25–800 ng/mL (Figure 9A,B). The LOD of SO in serum was 19.41 ng/mL, while that of CHA in serum was 16.92 ng/mL (Table 3). Further, the urinary SO and CHA could be measured in the concentration range of 50–800 ng/mL (Figure 9C,D). The LOD of SO in urine was 45.16 ng/mL, whereas that of CHA in urine was 38.15 ng/mL (Table 3). Based on these calibration curves, the simultaneous measurement of 16 samples of 100 ng/mL SO and CHA diluted in serum and urine greatly improved (Figure 9D,E and Table 3).

### 3.7. Construction of Calibration Curve for Simultaneous Measurement of SO and CHA

**Figure 10:**
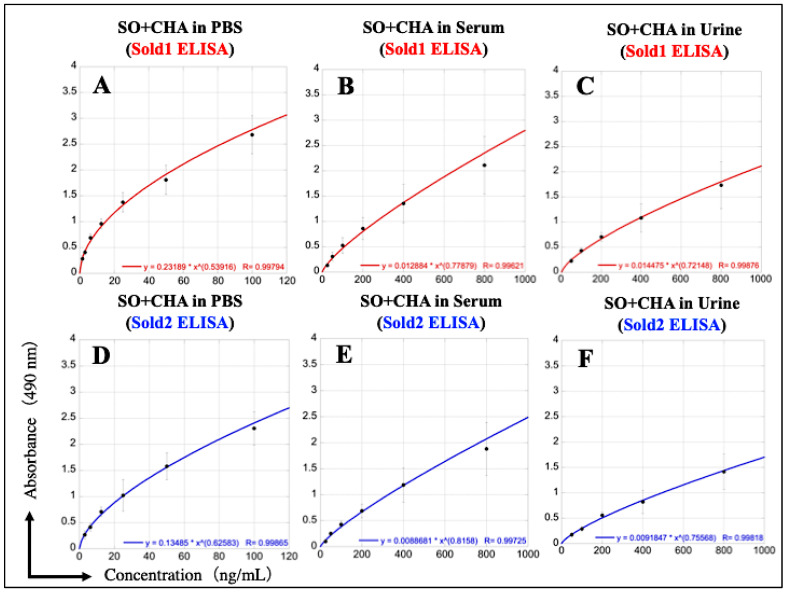
Calibration curves of each ELISA using serial dilutions with equal concentrations of the SO and CHA mixture in each matrix. The blank-subtracted absorbance mean (black circles) ± SD (error bars) values were plotted against SO and CHA mixture concentration to construct calibration curves by Sold1 ELISA (**A**–**C**) and Sold2 ELISA (**D**–**F**). In each graph, the *x*-axis indicates the SO and CHA concentration (ng/mL) in each mixture (e.g., “100” on the *x*-axis means that both the SO and CHA concentrations were 100 ng/mL in the mixture), whereas the *y*-axis indicates absorbance at 490 nm. The samples for all ELISAs were seven two-fold serial dilutions of the SO and CHA mixture standard, using PBS (1.56–100 ng/mL, **A**,**D**), serum (12.5–800 ng/mL, **B**,**E**), and urine (12.5–800 ng/mL, **C**,**F**) as the diluent and blank. The calibration curves were graphed, and the formula and R-value were calculated using the KaleidaGraph software. SO + CHA; SO and CHA were in equal concentrations in the mixture.

We examined whether it is possible to simultaneously measure both SO and CHA in each solvent. Good calibration curves could be drawn for SO and CHA samples diluted in PBS in the concentration range of 1.56–100 ng/mL in Sold1 ELISA and 3.12–100 ng/mL in Sold2 ELISA (Figure 10A,D). However, for the samples containing both SO and CHA diluted in serum, calibration curves could be constructed in the concentration range of 25–800 ng/mL in the two ELISAs (Figure 10B,E). Similarly, for urine samples containing both SO and CHA, calibration curves could be made in the concentration range of 50–800 ng/mL in the two ELISAs (Figure 10C,F).

### 3.8. Detection Performance of SO and CHA in Potato Extracts

**Figure 11:**
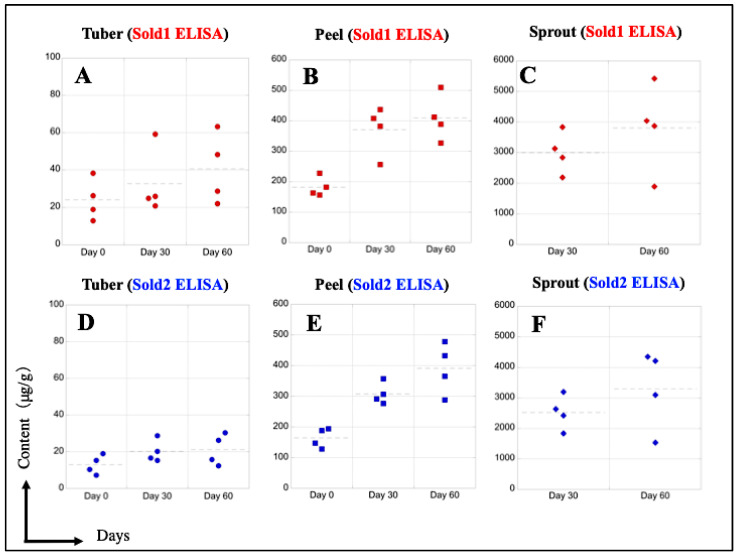
Quantification of SO and CHA concentrations in potato extracts using ELISA. Irish Cobbler potatoes (*n* = 4 on each Day) were used to prepare the extracts of each potato part. After measuring the concentrations of SO and CHA in each part extract, based on calibration curves constructed by PBS serial dilutions (Figure 10A,D), the content was calculated by multiplying their weights. The SO and CHA concentrations (μg/g) in tuber (**A**,**D**, circle), peel (**B**,**E**, square), and sprout (**C**,**F**, diamond), as calculated by Sold1 ELISA (**A**–**C**, red) and Sold2 ELISA (**D**–**F**, blue). In each graph, the *x*-axis indicates the elapsed period since potato purchase (Day 0, 30, and 60) and the *y*-axis indicates the content in each part (μg/g). The dotted lines indicate the average of the measurements.

The concentration of SO and CHA in each part of the potato was determined using ELISAs. In potato tubers, the SO and CHA content increased about 1.6- to 1.8-fold from Day 0 to Day 60 (Figure 11A,D). SO and CHA was detected in the peel extracts at about 180 ng/mL on Day 0 and increased to more than two-fold (~400 ng/mL) on Day 60 (Figure 11B,E). However, SO and CHA were detected in the extract of sprouts at about 3000 ng/mL on Day 30, and the same or higher levels of both chemicals were found on Day 60 (Figure 11C,F). In addition, no color development was observed in the samples prepared with distilled water at concentrations of 800 ng/mL of starch, pectin, glucose, and fructose, which are nutrients that are abundant in potatoes.

### 3.9. HPLC Analysis of SO and CHA in Potato Extracts

**Figure 12:**
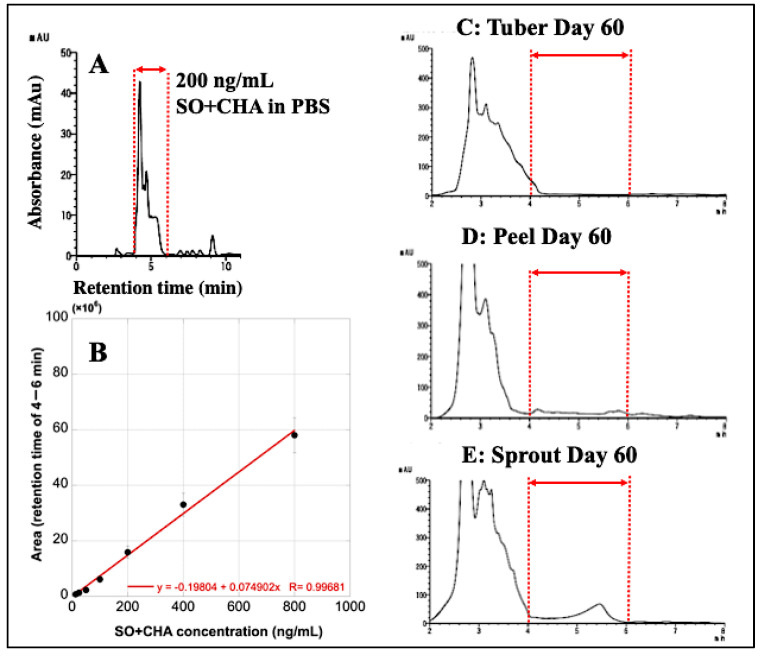
HPLC analysis of SO and CHA in PBS and potato extracts. A 200 ng/mL SO and CHA mixture diluted in PBS were analyzed by HPLC (**A**). The *x*-axis shows the retention time (min), and the *y*-axis shows the absorbance (mAu) at 210 nm. The waveforms at a retention time of 4–6 min (surrounded by red dotted lines) were determined to reflect mainly SO and CHA in solution. A calibration curve of HPLC using serial dilutions of equal concentrations of SO and CHA mixture in PBS (**B**). The calculated area’s mean (black circles) ± SD (error bars) values were plotted against SO and CHA mixture concentration to construct calibration curves by HPLC. The *x*-axis indicates the SO and CHA concentrations (ng/mL) in PBS, whereas the *y*-axis indicates the calculated areas under the waveform at a retention time of 4–6 min. The samples for HPLC analysis were 7 two-fold serial dilutions of the SO and CHA mixture standard using PBS (12.5–800 ng/mL). The calibration curve was graphed, and the formula and R-value were calculated using the KaleidaGraph software. The tuber (**C**), peel (**D**), and sprout (**E**) extracts derived from Irish Cobbler potatoes on Day 60 were analyzed by this HPLC. The *x*-axis shows the retention time (min), and the *y*-axis shows absorbance (mAu) at 210 nm. Retention times of 4–6 min, where SO and CHA are expected to be mainly detected, are delimited by red dotted lines. The area under the waveform detected in this range was matched with the calibration curve (**B**), and the calculated SO and CHA contents (μg/g) are shown in Table 4. SO + CHA, SO, and CHA are in equal concentrations in the mixture.

Finally, the concentration of SO and CHA in each part of the potato was analyzed by HPLC. When the mixture of SO and CHA dissolved in PBS was analyzed, their main waveforms were detected at a retention time of 4–6 min (Figure 12A). By using the relationship between the concentrations of SO and CHA in PBS and the area under the waveform at this retention time, a good calibration curve could be drawn in the concentration range of 12.5–800 ng/mL (Figure 12B). However, when the extracts from each potato part were measured with HPLC, no clear waveforms could be detected using a retention time of 4–6 min (Figure 12C–E). Therefore, the waveforms that were barely detected during this time period were used to calculate the SO and CHA contents of each potato part. Differences in the results between the ELISA and HPLC analyses of the SO and CHA calculated content for the same potato (*n* = 4) parts on Day 60 are shown in Table 4.

## 4. Discussion

Here, we successfully developed two new polyclonal antibodies that can bind to both SO and CHA and constructed two ELISA systems based on these antibodies. Although these antibodies are currently owned only by us, they can be produced by any institute by immunizing rabbits with the solanidine-BSA complex shown in Figure 2. Moreover, the immunization of mice with this solanidine-BSA complex may allow for the creation of monoclonal antibodies with better sensitivity and specificity for the detection of SO and CHA. Thus, our results provide essential information for all scientists involved in potato food poisoning research.

As potatoes contain both SO and CHA, developing antibodies that can bind to both SO and CHA would be the best way to construct an ELISA to detect them. Therefore, we aimed to establish antibodies that can recognize a part of solanidine, a chemical structure shared by SO and CHA, as epitopes. However, the molecular weight of solanidine is considerably low (400 Da), and the epitopes to which antibodies can bind are extremely limited. Since anti-sold1 and anti-sold2 in this study probably recognized almost the same epitope, we determined that it would be difficult to construct an analytical method using two different antibodies that recognize different epitopes, such as a sandwich ELISA system. Thus, we established non-competitive direct ELISA systems using anti-sold1 and anti-sold2. Both the ELISAs in our study were more sensitive to CHA than to SO, and the Sold1 ELISA appeared to have better detection performance than the Sold2 ELISA.

The constructed ELISAs in this study are comparable to the existing assays in terms of their ability to detect SO and CHA diluted in buffer solution. Our ELISA systems are about 20 times more sensitive in terms of LODs than previous ELISA kits that use monoclonal antibodies [10]. In particular, the LODs measuring SO and CHA in PBS by Sold1 ELISA are comparable to the results of previous HPLC analyses [8,11]. Although the Sold1 ELISA is not as sensitive as the combination of HPLC and mass spectrometry for SO and CHA [12], our ELISAs were good assays in terms of accuracy and precision, at least for the measurement of these chemicals diluted in PBS. Unfortunately, the sensitivity of our ELISA for SO and CHA in serum was low. A previous study reported an HPLC assay system that measured GAs (SO and CHA) in serum at a LOD of 0.3 ng/mL [13], which is about 15 times more sensitive than our ELISAs. In the same report, the peak serum concentrations after the ingestion of SO at 0.41 mg/kg body weight and CHA at 0.59 mg/kg body weight were 8 ng/mL and 14 ng/mL, respectively [13], which cannot be measured by our ELISAs. Thus, the detection performance of SO and CHA in serum needs to be improved in the future. In our experiment, when serum was used as the matrix, the blank containing only serum without SO or CHA showed higher absorbance, which may have contributed to the overall low detection sensitivity. This non-specific reaction may be caused by the strong influence of several components in the serum, especially the protein component. In fact, the addition of 10% trichloroacetic acid to the serum suppressed nonspecific coloration, suggesting that the deproteinization in serum samples is very important. Because of the great variety of proteins in serum, it would be difficult to narrow down the protein components that cross-react with antibodies, so an efficient protein removal method is desired. However, since the strong deproteinization also seemed to remove SO and CHA from the samples, a more careful examination is needed to optimize this process. A previous report suggested that immunoglobulins in the serum sporadically bind to each well of an ELISA plate and that the binding of HRP-labeled antibodies to these immunoglobulins causes false-positive reactions [24]. To solve this problem, the deproteinization of serum samples appears to be a good approach. However, the method of protein removal by denaturing protein components in serum with acids or organic solvents may affect SO and CHA. Since the protein removal method based on filtration is easy to apply, it is considered an ideal pre-treatment method for ELISA. Thus, there are many options for deproteinization, each of which has its own merits and demerits that cannot be verified in this study, but we would like to challenge them in the future. Because there are few previous reports on the measurement of SO and CHA in urine samples, comparing the detection sensitivity of our method with that of existing methods was impossible. However, in the present study, the LODs of SO and CHA were inferior in urine than in serum, and we must consider that the detection performance in urine was poor. When ELISA was performed, the coloration of urine samples was weaker than that of the PBS and serum samples, and the absorbance of the urine samples was lower than that of the other samples. This is probably due to the high concentration of urea in the urine samples. Since urea is known to act as a protein-denaturing agent [25], we speculated that the urea bound to each well of the ELISA plate denatured the HRP-labeled antibodies. Since a method to completely remove only urea from urine has not yet been established, we expect that it would be difficult to improve this detection performance of SO and CHA in urine. For both urine and serum, it would be desirable to prepare affinity columns conjugated with anti-sold1 or anti-sold2 and treat the samples with them to specifically separate SO and CHA from the biological components. Considering the CV and recovery, the variation and accuracy of SO and CHA diluted in serum or urine in ELISA are inferior to those of SO and CHA diluted in PBS. At present, it would be difficult to adapt our ELISA as a laboratory method for patients with potato food poisoning, but with further improvement, it may be feasible.

Although the Sold1 ELISA and Sold2 ELISA values were slightly different, the combined concentrations of SO and CHA could be measured from the extracts of each part of the potato. The SO and CHA concentrations in most tubers of commercial potatoes do not exceed 100 μg/g [26], whereas those in sprouts and peels are in the ranges of 2000–4000 μg/g and 300–600 μg/g, respectively [27]. Our experimental results were in the range of these concentrations or slightly higher. Each part of the potato contains not only SO and CHA but also solanidine and other GAs, and antibodies can bind to these chemicals as well, resulting in rather high values. In addition, it is also important to verify the cross-reactivity of antibodies with the components in potatoes other than GAs. Basically, 100 g of potato contains a very high amount of carbohydrates (21.44 g), with some fiber (2.3 g) and sugars (1.08 g) [28]. Our ELISAs did not react with starch (carbohydrates), pectin (fiber), and monosaccharides (sugars), suggesting that these assays effectively captured the toxic components in the potatoes. However, the accuracy of the results of the HPLC analysis is doubtful, since no clear waveforms indicating SO and CHA could be obtained from the potato extract samples. Since the contents of SO and CHA were underestimated by this HPLC analysis, this does not seem to be appropriate as a food testing method. It is not clear whether ELISA or HPLC analysis is more accurate, but given the unstable detection waveform of HPLC, ELISA seemed to have an advantage. As anti-sold1 and anti-sold2 bind to the substances with solanidine structure, it is necessary to further investigate the cross-reactivity of these polyclonal antibodies in detail. Although it would not reach the level of food poisoning, eating potatoes with a SO and CHA content exceeding 140 μg/g gives a bitter taste, and eating potatoes with SO and CHA content exceeding 220 μg/g contributes to a burning sensation in the mouth and throat [4,26]. To adopt our ELISAs as food testing methods, they need to be accurate enough to distinguish these subtle differences in SO and CHA concentration. We would like to re-examine each step of our ELISAs and develop a more accurate assay. Furthermore, it is important to re-establish competitive ELISA and indirect ELISA methods to enhance detection performance. In competitive ELISA, the antigens are pre-labeled, and samples containing a large amount of components other than the target chemicals can also be used [29]. As competitive ELISA requires only one type of antibody, it is relatively inexpensive and has been widely adopted in medical facilities. In contrast, the indirect ELISA method is cost-prohibitive as it uses a labeled secondary antibody in addition to the primary antibody, but it is expected to significantly improve the detection sensitivity [29]. In the future, we will attempt to construct ELISA methods based on these different principles so that researchers can customize the assay for different purposes and situations.

Non-competitive direct ELISAs are advantageous because several samples can be measured simultaneously on a single ELISA plate. In this respect, ELISA is superior to HPLC because the analysis of one sample must be completed before the analysis of the next sample can begin in the HPLC method. Another advantage of ELISA is that it does not require expensive equipment and is relatively easier to perform. ELISA only requires a microplate reader, which is less expensive than HPLC or mass spectrometry instruments. Moreover, optimizing anti-sold1 and anti-sold2 as medical reagents will enable their adaption to immunological analyzers that are already installed in medical facilities. Another merit of ELISA is the easy analysis of results—they can be interpreted visually as the concentrations of SO and CHA are indicated by the degree of coloration. The interpretation of HPLC and mass spectrometry results requires a certain degree of skills and expertise, and their use in medical facilities is limited. Notably, most of the previous analytical methods for SO and CHA were reported before 2000 AD [8,9,10,13], and there has been little development of detection methods for these two toxins in the last 20 years. Under such circumstances, our proposed method of creating polyclonal antibodies that bind to SO and CHA, and ELISAs that use these antibodies, possesses a high novelty. Even if only the two antibodies produced in this study are used, it is possible to construct new indirect ELISAs, sandwich ELISAs, and competitive ELISAs; moreover, further development would be expected as well. This increase in options for measuring SO and CHA is academically important and will provide useful evidence for many researchers involved in laboratory medicine and food testing.

ELISA also has some limitations, the primary one being that the measurement time currently takes more than one day. The first step of overnighting the samples on the plate is time-consuming, and a future challenge would be to shorten the measurement time. The second limitation is the high running cost. ELISA requires the use of HRP-conjugated antibodies for each assay, which are costlier than HPLC reagents. Additionally, detection sensitivity is a weak point when serum or urine is used as samples.

While there have been limited assay methods for SO and CHA, the development of non-competitive ELISAs is a useful achievement for the future development of potato food poisoning diagnosis and food testing. In the future, we aim to seek optimal conditions for ELISA with the goals of shortening the analysis time and improving sensitivity and specificity. Furthermore, we would like to start the construction of an immunochromatography using the antibodies developed in this study to establish a more rapid and simple method for measuring SO and CHA.

## 5. Conclusions

In this study, non-competitive direct ELISAs that can measure SO and CHA in both serum and urine samples were developed. However, the detection sensitivity remains an issue. Although cross-reactions with other components in the biological samples may occur, these ELISAs can measure SO and CHA in potato extracts. Therefore, these ELISAs would not be applicable to clinical testing but may be useful for food testing. The improvement of the sample preparation process and assay conditions can increase the applicability of these ELISAs in the future.

## Figures and Tables

**Table 1:** The detection performance of Sold1 ELISA based on its calibration curves obtained using PBS serial dilutions.

	Sold1 ELISA	100 (ng/mL)	50 (ng/mL)	10 (ng/mL)		Sold1 ELISA	100 (ng/mL)	50 (ng/mL)	10 (ng/mL)
SO in PBS	Mean (ng/mL)	98.49	51.74	10.89	CHA in PBS	Mean (ng/mL)	101.70	52.76	9.64
	SD	5.58	4.50	1.72		SD	5.62	3.45	1.32
	CV (%)	5.66	8.70	15.80		CV (%)	5.53	6.55	13.71
	Recovery (%)	97.46	95.20	89.20		Recovery (%)	105.28	101.58	98.20
	LOD (ng/mL)	1.38				LOD (ng/mL)	1.08		
SO in serum	Mean (ng/mL)	196.95	102.38		CHA in serum	Mean (ng/mL)	208.83	114.44	
	SD	16.85	14.30			SD	15.29	14.27	
	CV (%)	8.55	13.96			CV (%)	7.32	12.47	
	Recovery (%)	122.18	119.82			Recovery (%)	120.52	126.84	
SO in urine	Mean (ng/mL)	60.73	22.16		CHA in urine	Mean (ng/mL)	78.53	39.85	
	SD	5.14	3.77			SD	4.66	5.08	
	CV (%)	8.47	17.03			CV (%)	5.93	12.74	
	Recovery (%)	78.26	68.56			Recovery (%)	84.18	81.65	

Sixteen equivalent samples were used for each measurement condition.

**Table 2:** The detection performance of Sold2 ELISA based on its calibration curves obtained using PBS serial dilutions.

	Sold2 ELISA	100 (ng/mL)	50 (ng/mL)	10 (ng/mL)		Sold2 ELISA	100 (ng/mL)	50 (ng/mL)	10 (ng/mL)
SO in PBS	Mean (ng/mL)	101.01	49.23	8.14	CHA in PBS	Mean (ng/mL)	100.89	51.45	9.67
	SD	4.88	4.55	2.36		SD	3.63	4.22	1.88
	CV (%)	4.83	9.24	28.96		CV (%)	3.60	8.21	19.48
	Recovery (%)	102.25	97.68	90.18		Recovery (%)	101.95	104.25	96.42
	LOD (ng/mL)	2.95				LOD (ng/mL)	2.76		
SO in serum	Mean (ng/mL)	146.98	84.44		CHA in serum	Mean (ng/mL)	162.35	90.88	
	SD	12.63	13.26			SD	12.47	11.70	
	CV (%)	8.59	15.71			CV (%)	7.68	12.88	
	Recovery (%)	114.56	125.82			Recovery (%)	120.88	129.27	
SO in urine	Mean (ng/mL)	50.79	19.11		CHA in urine	Mean (ng/mL)	57.01	26.87	
	SD	4.54	3.65			SD	4.47	4.76	
	CV (%)	8.95	19.11			CV (%)	7.83	17.71	
	Recovery (%)	77.25	68.28			Recovery (%)	78.82	77.16	

Sixteen equivalent samples were used for each measurement condition.

**Table 3:** The detection performance of ELISAs based on the corrected calibration curves obtained using the serum and urine serial dilution measurements.

	Sold1 ELISA	100 (ng/mL)	Sold2 ELISA	100 (ng/mL)
SO in serum	Mean (ng/mL)	98.48	Mean (ng/mL)	96.55
	SD	8.42	SD	8.62
	CV (%)	8.55	CV (%)	8.92
	Recovery (%)	96.58	Recovery (%)	94.15
	LOD (ng/mL)	15.25	LOD (ng/mL)	19.41
SO in urine	Mean (ng/mL)	93.59	Mean (ng/mL)	102.20
	SD	8.65	SD	9.43
	CV (%)	9.24	CV (%)	9.23
	Recovery (%)	90.88	Recovery (%)	105.64
	LOD (ng/mL)	30.28	LOD (ng/mL)	45.16
CHA in serum	Mean (ng/mL)	103.60	Mean (ng/mL)	98.97
	SD	8.50	SD	9.29
	CV (%)	8.20	CV (%)	9.38
	Recovery (%)	106.62	Recovery (%)	96.67
	LOD (ng/mL)	13.48	LOD (ng/mL)	16.92
CHA in urine	Mean (ng/mL)	102.02	Mean (ng/mL)	103.69
	SD	7.97	SD	8.22
	CV (%)	7.81	CV (%)	7.93
	Recovery (%)	105.74	Recovery (%)	106.58
	LOD (ng/mL)	27.92	LOD (ng/mL)	38.15

**Table 4:** Differences between ELISA and HPLC analyses of the SO and CHA calculated content of the same potato parts on Day 60.

	Calculated Content (μg/g) Using Sold1 ELISA			
	(*n* = 4)	Tuber	Peel	Sprout
**Potato** **on** **Day 60**	**No.1**	22.04	389.0	1892
	**No.2**	63.28	510.2	3868
	**No.3**	48.20	327.1	5423
	**No.4**	28.68	412.4	4038
	**Calculated Content** (**μg/g**) **Using Sold2 ELISA**			
	(***n* = 4**)	**Tuber**	**Peel**	**Sprout**
**Potato** **on** **Day 60**	**No.1**	15.68	365.1	1538
	**No.2**	30.28	478.4	3102
	**No.3**	26.22	288.0	4348
	**No.4**	12.25	432.8	4212
	**Calculated Content** (**μg/g**) **Using HPLC**			
	(***n* = 4**)	**Tuber**	**Peel**	**Sprout**
**Potato** **on** **Day 60**	**No.1**	8.27	170.3	1065
	**No.2**	13.95	209.8	1982
	**No.3**	10.16	152.7	2643
	**No.4**	8.66	202.0	2129

## Data Availability

The data presented in this study are available in this article.
